# Supplementary material for: Circular RNA repertoires are associated with evolutionarily young transposable elements
Source: eLife. 2021 Sep 20;10:e67991. doi: 10.7554/eLife.67991 (PMC8516420; doi:10.7554/eLife.67991)
Supplement: Supplementary file 1. — Summary of organism, tissue, age and sex for each sample; last column shows the RNA Quality Number (RQN) for the extracted RNA. [file elife-67991-supp1.docx]

###### **Supplementary File 1: Sample overview.**

**Supplementary File 1.** Summary of organism, tissue, age and sex for each sample; last column shows the RNA Quality Number (RQN) for the extracted RNA.

| **Species** | **Tissue** | **Age** | **Sex** | **RQN** |
| --- | --- | --- | --- | --- |
| Opossum | Cerebellum | 21 months | male | 7.3 |
| Opossum | Cerebellum | 19.5 months | male | 8.9 |
| Opossum | Cerebellum | 15.5 months | male | 6.8 |
| Opossum | Liver | 15.5 months | male | 9.3 |
| Opossum | Liver | 21 months | male | 8.6 |
| Opossum | Liver | 13 months | male | 9 |
| Opossum | Testis | 21 months | male | 8.9 |
| Opossum | Testis | 13 months | male | 8.5 |
| Opossum | Testis | 15.5 months | male | 8.9 |
| Mouse | Cerebellum | 9 weeks | male | 7.1 |
| Mouse | Cerebellum | 9 weeks | male | 7.4 |
| Mouse | Cerebellum | 9 weeks | male | 7 |
| Mouse | Liver | 9 weeks | male | 7.9 |
| Mouse | Liver | 9 weeks | male | 7.6 |
| Mouse | Liver | 9 weeks | male | 8.5 |
| Mouse | Testis | 9 weeks | male | 8.4 |
| Mouse | Testis | 9 weeks | male | 8.2 |
| Mouse | Testis | 9 weeks | male | 8.4 |
| Rat | Cerebellum | 16 weeks | male | 7.2 |
| Rat | Cerebellum | 16 weeks | male | 7.5 |
| Rat | Cerebellum | 16 weeks | male | 7.7 |
| Rat | Liver | 16 weeks | male | 7.2 |
| Rat | Liver | 16 weeks | male | 7.9 |
| Rat | Liver | 16 weeks | male | 7.8 |
| Rat | Testis | 16 weeks | male | 7.7 |
| Rat | Testis | 16 weeks | male | 8.8 |
| Rat | Testis | 16 weeks | male | 7.8 |
| Rhesus macaque | Cerebellum | 8 years | male | 8.5 |
| Rhesus macaque | Cerebellum | 9 years | male | 7.7 |
| Rhesus macaque | Liver | 8 years | male | 8.6 |
| Rhesus macaque | Liver | 9 years | male | 8.2 |
| Rhesus macaque | Liver | 9 years | male | 8.6 |
| Rhesus macaque | Testis | 8 years | male | 9.5 |
| Rhesus macaque | Testis | 9 years | male | 9.1 |
| Rhesus macaque | Testis | 8 years | male | 8.8 |
| Human | Liver | 64 years | male | 7.5 |
| Human | Cerebellum | 29 years | male | 8.2 |
| Human | Cerebellum | 41 years | male | 8.6 |
| Human | Cerebellum | 25 years | male | 8.3 |
| Human | Testis | 21 years | male | 7.8 |
| Human | Testis | 41 years | male | 6.9 |
| Human | Testis | 22 years | male | 6.9 |
