# Supplementary material for: Circular RNA repertoires are associated with evolutionarily young transposable elements
Source: eLife. 2021 Sep 20;10:e67991. doi: 10.7554/eLife.67991 (PMC8516420; doi:10.7554/eLife.67991)
Supplement: Supplementary file 2. — Description of the different filtering steps applied to generate a high confidence circRNA dataset based on the comparison of untreated and RNase R-treated samples. The number of unique BSJs left after each filtering step is shown for each tissue (see Materials and methods, section Generation of high confidence circRNA candidates from the comparison of RNase R-treated vs. -untreated samples); mouse was chosen as representative example. [file elife-67991-supp2.docx]

###### **Supplementary File 2: Filtering steps and reduction of circRNAs candidates during the identification pipeline.**

**Supplementary File 2.** Description of the different filtering steps applied to generate a high confidence circRNA dataset based on the comparison of untreated and RNase R-treated samples. The number of unique BSJs left after each filtering step is shown for each tissue (see **Material and Methods,** section *Generation of high confidence circRNA candidates from the comparison of RNase R-treated vs. -untreated samples*); mouse was chosen as representative example.

|  | **Liver** | **Cerebellum** | **Testis** |
| --- | --- | --- | --- |
| After read mapping, the lists of BSJs in untreated and RNase R treated was merged for each biological replicate keeping all BSJs that were detected in either the untreated or the RNase R-treated sample. The total number of unique BSJs in each biological replicate is shown together with the number of unique BSJs in the untreated and RNase R-treated biological replicate. | | | |
| Biological replicate 1  (untreated \| RNAse R) | 24,474  (4,483 \| 20,674) | 55,455  (15,409 \| 45,454) | 47,794  (9,491 \| 42,362) |
| Biological replicate 2  (untreated \| RNAse R) | 26,575  (4,788 \| 22,602) | 52,229  (13,724 \| 48,322) | 36,843  (9,427 \| 30,590) |
| Biological replicate 3  (untreated \| RNAse R) | 23,699  (5,111 \| 19,357) | 68,154  (18,510 \| 56,725) | 40,907  (6,063 \| 37,347) |
| **Filtering step 1**  When mapping paired-end sequencing data, both reads should ideally map to the genome (paired-end = “pe”). However, sometimes one of the mate reads cannot be mapped due to the complexity of the genomic locus. These reads are reported as “singletons” (“se”). We only kept BSJs for which both read mates mapped consistently either in “pe” or “se” mode (see **Material and Methods** for more details).  The number of BSJs in each sample, which remain after filtering step 1, are indicated. | | | |
| Biological replicate 1  (% kept after filtering step 1) | 24,373  (99.59%) | 54,840  (98.89%) | 47,416  (99.21%) |
| Biological replicate 2  (% kept after filtering step 1) | 26,502  (99.73%) | 51,725  (99.00%) | 36,439  (98.90%) |
| Biological replicate 3  (% kept after filtering step 1) | 23,568  (99.57%) | 67,370  (98.85%) | 40,544  (99.11%) |
| Total number of unique BSJs across all samples (untreated and RNase R-treated) | 66,405 | 137,615 | 94,831 |
| **Filtering step 2**  We assume that to have some kind of potential function, circRNAs need to be present in normal conditions. We thus removed all BSJs which were only present in RNase R treated samples and could not be detected in any of the untreated, biological replicates.  The number of unique BSJs, which remain after filtering step 2, are indicated. | | | |
| Total number of unique BSJs across all samples  (% kept from total, unique BSJs after filtering step 2) | 13,084  (19.70%) | 37,086  (26.95%) | 20,358  (21.47%) |
| **Filtering step 3**  Next, BSJs were normalized by the size factor of each sample (see **Material and Methods**) and the mean, normalised count was calculated for each condition (untreated and RNase R treated). Next, the log2-enrichment for RNase R-treated vs. -untreated samples was calculated. All BSJs for which the log2-enrichment was below 1.5 were removed.  The number of BSJs in all untreated samples, which remain after filtering step 3, are indicated. | | | |
| Total number of unique BSJs across all samples  (% kept from total, unique BSJs after filtering step 3) | 1,914  (2.88%) | 8,139  (5.91%) | 6,381  (6.73%) |
| **Filtering step 4**  The mean RPM value for each BSJ across untreated replicates was calculated. All BSJs with at least 0.05 were kept. These loci were considered strong circRNA candidates and used for all subsequent analyses.  The final number of circRNAs, which remain after filtering step 4, are indicated. | | | |
| Total number of unique BSJs across all samples = final circRNA candidates  (% kept from total, unique BSJs after filtering step 4) | 87  (0.13%) | 1,054  (0.77%) | 523  (0.55%) |

###### 
