# Supplementary material for: Circular RNA repertoires are associated with evolutionarily young transposable elements
Source: eLife. 2021 Sep 20;10:e67991. doi: 10.7554/eLife.67991 (PMC8516420; doi:10.7554/eLife.67991)
Supplement: Supplementary file 3. — Table summarises the total number of detected BSJs after the filtering step in each species. The percentage of BSJs that are unique to one, two, three or more than three samples of the same species is shown. [file elife-67991-supp3.docx]

###### **Supplementary File 3: Detected back splice junctions (BSJs) across samples.**

**Supplementary File 3.** Table summarises the total number of detected BSJs after the filtering step in each species. The percentage of BSJs that are unique to one, two, three or more than three samples of the same species is shown.

| **Species** | **Total BSJs** | **1 replicate** | **2 replicates** | **3 replicates** | **>= 4 replicates** |
| --- | --- | --- | --- | --- | --- |
| Opossum | 76,739 | 84.74 | 8.05 | 4.28 | 2.93 |
| Mouse | 67,249 | 83.45 | 9.23 | 4.73 | 2.59 |
| Rat | 72,855 | 85.43 | 7.73 | 3.88 | 2.96 |
| Rhesus macaque | 100,270 | 79.29 | 9.79 | 4.83 | 6.09 |
| Human | 68,400 | 79.86 | 10.71 | 6.54 | 2.9 |
