# Supplementary material for: Circular RNA repertoires are associated with evolutionarily young transposable elements
Source: eLife. 2021 Sep 20;10:e67991. doi: 10.7554/eLife.67991 (PMC8516420; doi:10.7554/eLife.67991)
Supplement: Supplementary file 4. — Indicated is the total number of different circRNAs that were annotated in each of the tissues across species. [file elife-67991-supp4.docx]

###### **Supplementary File 4: Total number of circRNAs in different species and tissues.**

**Supplementary File 4.** Indicated is the total number of different circRNAs that were annotated in each of the tissues across all species.

| **Species** | **Liver** | **Cerebellum** | **Testis** |
| --- | --- | --- | --- |
| Opossum | 129 | 417 | 1229 |
| Mouse | 87 | 1054 | 523 |
| Rat | 114 | 996 | 1192 |
| Rhesus macaque | 601 | 2132 | 1367 |
| Human | 765 | 2994 | 1761 |
