# Supplementary material for: Circular RNA repertoires are associated with evolutionarily young transposable elements
Source: eLife. 2021 Sep 20;10:e67991. doi: 10.7554/eLife.67991 (PMC8516420; doi:10.7554/eLife.67991)
Supplement: Supplementary file 5. — Spearman’s rank correlation for the GC amplitude and GC content of introns and exons are calculated for each isochore and species. The mean correlation between the GC amplitude and GC content of introns and exons is shown for different splice sites relative to the circRNA. [file elife-67991-supp5.docx]

###### **Supplementary File 5: Mean amplitude correlations.**

**Supplementary File 5.** Spearman’s rank correlation for the GC amplitude and GC content of introns and exons are calculated for each isochore and species. The mean correlation between the GC amplitude and GC content of introns and exons is shown for different splice sites relative to the circRNA.

| **Position** | **Amplitude ~ Intron** | **Amplitude ~ Exon** |
| --- | --- | --- |
| Non-parental | -0.42 | 0.31 |
| Outside of circRNA | -0.44 | 0.16 |
| Inside of circRNA | -0.48 | 0.40 |

###### 
