# Supplementary material for: Circular RNA repertoires are associated with evolutionarily young transposable elements
Source: eLife. 2021 Sep 20;10:e67991. doi: 10.7554/eLife.67991 (PMC8516420; doi:10.7554/eLife.67991)
Supplement: Supplementary file 6. — A generalised linear model was fitted to predict the probability of coding genes to be a parental gene (nopossum = 18807, nmouse = 22015, nrat = 11654, nrhesus = 21891, nhuman = 21744). The model was trained on 80% of the data (scaled values, cross-validation, 1000 repetitions, shown in rows labeled as ‘prediction’). Only the best predictors were kept and then used to predict probabilities for the remaining 20% of data points (validation set, shown in rows labeled as ‘validation’). Log-odds ratios, standard error and 95% confidence intervals (CI) for the validation set have been (beta) standardised. [file elife-67991-supp6.docx]

###### **Supplementary File 6: GLM summary for presence of parental genes.**

**Supplementary File 6.** A generalised linear model was fitted to predict the probability of coding genes to be a parental gene (n_opossum_ = 18,807, n_mouse_ = 22,015, n_rat_ = 11,654, n_rhesus_ = 21,891, n_human_ = 21,744). The model was trained on 80% of the data (scaled values, cross-validation, 1000 repetitions, shown in rows labeled as “prediction”). Only the best predictors were kept and then used to predict probabilities for the remaining 20% of data points (validation set, shown in rows labeled as “validation”). Log-odds ratios, standard error and 95% confidence intervals (CI) for the validation set have been (beta) standardised*.*

######

| **Predictor** | **Coefficient** | **Std. error** | **Lower CI** | **Upper CI** | **p-value** | **Species** | **Dataset** |
| --- | --- | --- | --- | --- | --- | --- | --- |
| as.rvc | 0.4282 | 0.0318 | 0.3658 | 0.4906 | 2.93E-41 | opossum | prediction |
| exon_count | 0.3267 | 0.0309 | 0.2661 | 0.3872 | 3.51E-26 | opossum | prediction |
| mean_brawand | 0.3314 | 0.0484 | 0.2367 | 0.4263 | 7.28E-12 | opossum | prediction |
| percentage_gc_content | -1.9481 | 0.1133 | -2.1751 | -1.7307 | 3.24E-66 | opossum | prediction |
| as.rvc | 0.2571 | 0.0307 | 0.1963 | 0.3168 | 5.54E-17 | mouse | prediction |
| exon_count | 0.3831 | 0.0318 | 0.3206 | 0.4454 | 2.14E-33 | mouse | prediction |
| percentage_gc_content | -0.8193 | 0.058 | -0.9341 | -0.7068 | 2.44E-45 | mouse | prediction |
| phastcons | 0.5777 | 0.0607 | 0.4613 | 0.6993 | 1.71E-21 | mouse | prediction |
| exon_count | 0.2199 | 0.0357 | 0.1495 | 0.2895 | 6.91E-10 | rat | prediction |
| genomic_length | 0.2624 | 0.0325 | 0.1985 | 0.3263 | 7.36E-16 | rat | prediction |
| mean_cpm | 0.2696 | 0.0489 | 0.174 | 0.3658 | 3.58E-08 | rat | prediction |
| percentage_gc_content | -0.5576 | 0.0601 | -0.6763 | -0.4408 | 1.68E-20 | rat | prediction |
| phastcons | 0.6314 | 0.0797 | 0.4802 | 0.793 | 2.35E-15 | rat | prediction |
| ss.rvc | 0.158 | 0.0416 | 0.0737 | 0.2373 | 0.000148111 | rat | prediction |
| as.rvc | 0.5653 | 0.0333 | 0.5001 | 0.6306 | 1.23E-64 | rhesus | prediction |
| exon_count | 0.3766 | 0.029 | 0.3197 | 0.4335 | 1.84E-38 | rhesus | prediction |
| genomic_length | 0.2506 | 0.026 | 0.2001 | 0.3022 | 6.36E-22 | rhesus | prediction |
| mean_brawand | 0.3162 | 0.0366 | 0.2446 | 0.3879 | 5.12E-18 | rhesus | prediction |
| percentage_gc_content | -1.3246 | 0.0586 | -1.4412 | -1.2114 | 4.06E-113 | rhesus | prediction |
| exon_count | 0.3848 | 0.0291 | 0.3279 | 0.4419 | 5.10E-40 | human | prediction |
| genomic_length | 0.1772 | 0.0254 | 0.1279 | 0.2274 | 2.87E-12 | human | prediction |
| mean_brawand | 0.2675 | 0.0359 | 0.197 | 0.3378 | 9.71E-14 | human | prediction |
| percentage_gc_content | -1.333 | 0.056 | -1.4442 | -1.2247 | 2.04E-125 | human | prediction |
| phastcons | 0.3218 | 0.0349 | 0.2538 | 0.3906 | 2.91E-20 | human | prediction |
| ss.rvc | 0.6142 | 0.0328 | 0.55 | 0.6787 | 3.25E-78 | human | prediction |
| exon_count | 0.4473 | 0.0646 | 0.3206 | 0.574 | 4.49E-12 | opossum | validation |
| percentage_gc_content | -1.8437 | 0.2168 | -2.2686 | -1.4188 | 1.82E-17 | opossum | validation |
| mean_brawand | 0.343 | 0.0961 | 0.1547 | 0.5313 | 0.000357262 | opossum | validation |
| as.rvc | 0.284 | 0.0656 | 0.1554 | 0.4127 | 1.51E-05 | opossum | validation |
| exon_count | 0.3757 | 0.0682 | 0.242 | 0.5095 | 3.65E-08 | mouse | validation |
| percentage_gc_content | -1.0861 | 0.1291 | -1.3391 | -0.8331 | 3.96E-17 | mouse | validation |
| as.rvc | 0.1967 | 0.063 | 0.0732 | 0.3202 | 0.001801116 | mouse | validation |
| phastcons | 0.5802 | 0.1226 | 0.3398 | 0.8205 | 2.24E-06 | mouse | validation |
| genomic_length | 0.2603 | 0.0727 | 0.1179 | 0.4027 | 0.000340157 | rat | validation |
| exon_count | 0.296 | 0.0732 | 0.1526 | 0.4395 | 5.24E-05 | rat | validation |
| percentage_gc_content | -0.7197 | 0.1252 | -0.9651 | -0.4743 | 9.02E-09 | rat | validation |
| mean_cpm | 0.1467 | 0.0982 | -0.0458 | 0.3392 | 0.135228403 | rat | validation |
| ss.rvc | 0.0848 | 0.0873 | -0.0863 | 0.2559 | 0.33133768 | rat | validation |
| phastcons | 0.5127 | 0.1478 | 0.223 | 0.8024 | 0.00052204 | rat | validation |
| genomic_length | 0.1716 | 0.0491 | 0.0754 | 0.2678 | 0.000474304 | rhesus | validation |
| exon_count | 0.415 | 0.0595 | 0.2984 | 0.5315 | 3.02E-12 | rhesus | validation |
| percentage_gc_content | -1.4385 | 0.121 | -1.6757 | -1.2013 | 1.39E-32 | rhesus | validation |
| mean_brawand | 0.3781 | 0.0722 | 0.2366 | 0.5197 | 1.64E-07 | rhesus | validation |
| as.rvc | 0.5888 | 0.0652 | 0.461 | 0.7165 | 1.67E-19 | rhesus | validation |
| genomic_length | 0.2624 | 0.0557 | 0.1533 | 0.3716 | 2.46E-06 | human | validation |
| exon_count | 0.3209 | 0.0613 | 0.2007 | 0.4411 | 1.67E-07 | human | validation |
| percentage_gc_content | -1.4173 | 0.1224 | -1.6572 | -1.1774 | 5.37E-31 | human | validation |
| mean_brawand | 0.2475 | 0.0773 | 0.096 | 0.3989 | 0.001363255 | human | validation |
| ss.rvc | 0.5809 | 0.0692 | 0.4453 | 0.7166 | 4.76E-17 | human | validation |
| phastcons | 0.453 | 0.0763 | 0.3034 | 0.6025 | 2.89E-09 | human | validation |

###### 
