# Supplementary material for: Circular RNA repertoires are associated with evolutionarily young transposable elements
Source: eLife. 2021 Sep 20;10:e67991. doi: 10.7554/eLife.67991 (PMC8516420; doi:10.7554/eLife.67991)
Supplement: Supplementary file 7. — A generalised linear model was fitted to predict the probability of a hotspot to be present across multiple species (nopossum = 872, nmouse = 848, nrat = 665, nrhesus = 1682, nhuman = 2022). Reported log-odds ratios, standard error and 95% confidence intervals (CI) are (beta) standardised. [file elife-67991-supp7.docx]

###### **Supplementary File 7: GLM summary for “sharedness” of hotspots.**

**Supplementary File 7.** A generalised linear model was fitted to predict the probability of a hotspot to be present across multiple species (n_opossum_ = 872, n_mouse_ = 848, n_rat_ = 665, n_rhesus_ = 1,682, n_human_ = 2,022). Reported log-odds ratios, standard error and 95% confidence intervals (CI) are (beta) standardised.

| **Predictor** | **Coefficient** | **Std. error** | **Lower CI** | **Upper CI** | **p-value** | **Species** |
| --- | --- | --- | --- | --- | --- | --- |
| therian | 0.4283 | 0.0796 | 0.2723 | 0.5843 | 7.40E-08 | opossum |
| rodents | 0.2883 | 0.0909 | 0.11 | 0.4665 | 0.001525767 | mouse |
| eutherian | 0.6723 | 0.0981 | 0.4801 | 0.8646 | 7.10E-12 | mouse |
| therian | 0.7228 | 0.0882 | 0.5499 | 0.8956 | 2.49E-16 | mouse |
| rodents | 0.2048 | 0.0954 | 0.0178 | 0.3918 | 0.031813121 | rat |
| eutherian | 0.5835 | 0.0997 | 0.3881 | 0.779 | 4.87E-09 | rat |
| therian | 0.7539 | 0.0916 | 0.5744 | 0.9335 | 1.88E-16 | rat |
| primates | 0.4241 | 0.0617 | 0.3032 | 0.545 | 6.07E-12 | rhesus |
| eutherian | 0.5736 | 0.0577 | 0.4606 | 0.6867 | 2.59E-23 | rhesus |
| therian | 0.4952 | 0.0563 | 0.3848 | 0.6056 | 1.49E-18 | rhesus |
| primates | 0.4065 | 0.0506 | 0.3073 | 0.5056 | 9.12E-16 | human |
| eutherian | 0.4564 | 0.0492 | 0.36 | 0.5527 | 1.65E-20 | human |
| therian | 0.6161 | 0.051 | 0.5162 | 0.7161 | 1.35E-33 | human |

######

###### 
