# Supplementary material for: Circular RNA repertoires are associated with evolutionarily young transposable elements
Source: eLife. 2021 Sep 20;10:e67991. doi: 10.7554/eLife.67991 (PMC8516420; doi:10.7554/eLife.67991)
Supplement: Supplementary file 8. — A generalised linear model was fitted to predict the probability of circRNA hotspots among parental genes; parental genes were filtered for circRNAs that were either species-specific or occurred in orthologous loci across therian species (nopossum = 869, nmouse = 503, nrat = 425, nrhesus = 912, nhuman = 1213). The model was trained on 80% of the data (scaled values, cross-validation, 1000 repetitions, shown in rows labeled as ‘prediction’). Only the best predictors were kept and then used to predict probabilities for the remaining 20% of data points (validation set, shown in rows labeled as ‘validation’). Log-odds ratios, standard error and 95% confidence intervals (CI) for the validation set have been (beta) standardised. [file elife-67991-supp8.docx]

###### **Supplementary File 8: GLM summary for circRNA hotspots among parental genes.**

######

**Supplementary File 8**. A generalised linear model was fitted to predict the probability of circRNA hotspots among parental genes; parental genes were filtered for circRNAs that were either species-specific or occurred in orthologous loci across therian species (n_opossum_ = 869, n_mouse_ = 503, n_rat_ = 425, n_rhesus_ = 912, n_human_ = 1,213). The model was trained on 80% of the data (scaled values, cross-validation, 1000 repetitions, shown in rows labeled as “prediction”). Only the best predictors were kept and then used to predict probabilities for the remaining 20% of data points (validation set, shown in rows labeled as “validation”). Log-odds ratios, standard error and 95% confidence intervals (CI) for the validation set have been (beta) standardised.

| **Predictor** | **Coefficient** | **Std. error** | **Lower CI** | **Upper CI** | **p-value** | **Species** | **Dataset** |
| --- | --- | --- | --- | --- | --- | --- | --- |
| percentage_gc_content | -1.27 | 0.3557 | -2.0031 | -0.6096 | 0.000357104 | opossum | prediction |
| percentage_gc_content | -0.5314 | 0.2027 | -0.9434 | -0.1466 | 0.008758284 | mouse | prediction |
| percentage_gc_content | -0.5665 | 0.1901 | -0.9536 | -0.2066 | 0.00287308 | rat | prediction |
| percentage_gc_content | -0.3979 | 0.1552 | -0.7119 | -0.1024 | 0.01035429 | rhesus | prediction |
| as.rvc | 0.3618 | 0.0882 | 0.1896 | 0.5359 | 4.12E-05 | human | prediction |
| percentage_gc_content | -0.9583 | 0.1558 | -1.2734 | -0.6622 | 7.63E-10 | human | prediction |
| percentage_gc_content | -1.438 | 0.4137 | -2.2489 | -0.6271 | 0.000509099 | opossum | validation |
| percentage_gc_content | -0.4325 | 0.2781 | -0.9776 | 0.1126 | 0.119942469 | mouse | validation |
| percentage_gc_content | -0.643 | 0.3373 | -1.3042 | 0.0182 | 0.056634202 | rat | validation |
| percentage_gc_content | -0.4345 | 0.198 | -0.8226 | -0.0463 | 0.028234012 | rhesus | validation |
| percentage_gc_content | -0.4319 | 0.1693 | -0.7636 | -0.1001 | 0.010729656 | human | validation |
| as.rvc | 0.2547 | 0.1477 | -0.0347 | 0.5441 | 0.084501745 | human | validation |

###### 
