# Supplementary material for: Circular RNA repertoires are associated with evolutionarily young transposable elements
Source: eLife. 2021 Sep 20;10:e67991. doi: 10.7554/eLife.67991 (PMC8516420; doi:10.7554/eLife.67991)
Supplement: Supplementary file 9. — Highly expressed circRNAs were defined as the circRNAs present in the 90% expression quantile of a tissue in a species. Per species, the circRNAs in the 90% expression quantiles from each of the three tissues were then pooled for further analysis (nopossum = 158, nmouse = 156, nrat = 217, nrhesus = 340, nhuman = 471) and their properties compared to circRNAs outside the 90% expression quantile. Highly expressed circRNAs are designated ‘1’, others ‘0’. Differences in genomic length, circRNA length, exon number and GLM model performance were assessed with a Student’s t-Test; p-values are indicated in the table (ns = non-significant). [file elife-67991-supp9.docx]

###### **Supplementary File 9: Analysis of highly expressed circRNAs.**

**Supplementary File 9.** Highly expressed circRNAs were defined as the circRNAs present in the 90% expression quantile of a tissue in a species. Per species, the circRNAs in the 90% expression quantiles from each of the three tissues were then pooled for further analysis (n_opossum_ = 158, n_mouse_ = 156, n_rat_ = 217, n_rhesus_ = 340, n_human_ = 471) and their properties compared to circRNAs outside the 90% expression quantile. Highly expressed circRNAs are designated “1”, others “0”. Differences in genomic length, circRNA length, exon number and GLM model performance were assessed with a Student's t-Test; p-values are indicated in the table (ns = non-significant).

| **Property** | **Opossum** | **Mouse** | **Rat** | **Rhesus** | **Human** |
| --- | --- | --- | --- | --- | --- |
| Genomic length | ns | ns | ns | *p = 0.0043* | *p = 0.047* |
| circRNA length | ns | ns | ns | ns | ns |
| Exon number | ns | ns | ns | ns | *p < 0.001* |
| % of circRNAs expressed in all 3 tissues analysed (1 = highly expressed, 0 = others); more details in **Figure 3-Figure supplement 5A** | 0: 2.32%  1: 3.80% | 0: 0.82%  1: 8.97% | 0: 0.88%  1: 6.45% | 0: 4.22%  1: 15.88% | 0: 4.35%  1: 12.31% |
| % of circRNAs detected in a hotspot (1 = highly expressed, 0 = others); more details in **Figure 3-Figure supplement 5B** | 0: 37.33%  1: 53.16% | 0: 44.95%  1: 67.95% | 0: 51.07%  1: 71.89% | 0: 51.92%  1: 66.18% | 0: 57.06%  1: 72.61% |
| Median number of circRNAs present in hotspots with at least 1 (= 1) or no (= 0) highly expressed circRNA | 0: 3  1: 3 | 0: 3  1: 3 | 0: 3  1: 4.5 | 0: 3  1: 3 | 0: 3  1: 3 |
| Comparison of GLM model performance between parental genes with and without a highly expressed circRNAs | *p = 0.0163*  **Note:** GLM prediction values are higher (driven by a lower GC content) | *ns* | *ns* | *p = 0.05*  **Note:** GLM prediction values are higher (driven by genomic length, GC content and exon count) | *p < 0.001*  **Note:** GLM prediction values are higher (driven by genomic length, GC content and exon count) |
| Are highly expressed circRNAs more likely to be shared across species?  More details in  **Figure 3-Figure supplement 5C** and **Supplementary File 10** | Yes | Yes | Yes | Yes | Yes |
