# Supplementary material for: Circular RNA repertoires are associated with evolutionarily young transposable elements
Source: eLife. 2021 Sep 20;10:e67991. doi: 10.7554/eLife.67991 (PMC8516420; doi:10.7554/eLife.67991)
Supplement: Supplementary file 10. — A generalised linear model was fitted on the complete dataset to predict the probability of parental genes of highly expressed circRNAs to be produce circRNAs in multiple species (nopossum = 869, nmouse = 844, nrat = 661, nrhesus = 1673, nhuman = 2016). The ‘sharedness’ definition is based on the phylogeny of species as: present in only one species, in rodents (mouse, rat) or primates (rhesus, human), eutherian species (rodents + at least one primate, or primates + at least one rodent) and therian species (opossum + rodents + at least one primate, or opossum + primates + at least one rodents). Log-odds ratios, standard error, 95% confidence intervals (CI) and p-values are shown. [file elife-67991-supp10.docx]

###### **Supplementary File 10: GLM for highly expressed circRNAs based on ‘age groups’.**

**Supplementary File 10.** A generalised linear model was fitted on the complete dataset to predict the probability of parental genes of highly expressed circRNAs to be produce circRNAs in multiple species (n_opossum_ = 869, n_mouse_ = 844, n_rat_ = 661, n_rhesus_ = 1,673, n_human_ = 2,016). The “sharedness” definition is based on the phylogeny of species as: present in only one species, in rodents (mouse, rat) or primates (rhesus, human), eutherian species (rodents + at least one primate, or primates + at least one rodent) and therian species (opossum + rodents + at least one primate, or opossum + primates + at least one rodents). Log-odds ratios, standard error, 95% confidence intervals (CI) and p-values are shown.

| **Predictor** | **Coefficient** | **Std. Error** | **Lower CI** | **Upper CI** | **p-value** | **Species** |
| --- | --- | --- | --- | --- | --- | --- |
| therian | 0.9262 | 0.2171 | 0.4981 | 1.3513 | 2.00E-05 | opossum |
| eutherian | 1.1189 | 0.295 | 0.5526 | 1.7156 | 0.000148951 | mouse |
| rodents | 1.2415 | 0.3833 | 0.4708 | 1.9859 | 0.001199369 | mouse |
| therian | 1.7822 | 0.3092 | 1.1861 | 2.4045 | 8.22E-09 | mouse |
| eutherian | 1.1828 | 0.3223 | 0.5608 | 1.8324 | 0.000242748 | rat |
| rodents | 1.189 | 0.4794 | 0.189 | 2.0953 | 0.01312791 | rat |
| therian | 1.6279 | 0.359 | 0.9239 | 2.3407 | 5.77E-06 | rat |
| eutherian | 1.729 | 0.2151 | 1.3129 | 2.1582 | 9.11E-16 | rhesus |
| primates | 1.1084 | 0.2077 | 0.7074 | 1.5237 | 9.45E-08 | rhesus |
| etherian | 1.7435 | 0.2261 | 1.3039 | 2.1925 | 1.25E-14 | rhesus |
| eutherian | 1.3691 | 0.1818 | 1.0127 | 1.7266 | 5.08E-14 | human |
| primates | 1.1663 | 0.1671 | 0.8406 | 1.4966 | 2.97E-12 | human |
| therian | 1.782 | 0.1884 | 1.4131 | 2.1525 | 3.06E-21 | human |
