# Supplementary material for: Circular RNA repertoires are associated with evolutionarily young transposable elements
Source: eLife. 2021 Sep 20;10:e67991. doi: 10.7554/eLife.67991 (PMC8516420; doi:10.7554/eLife.67991)
Supplement: Supplementary file 11. — The total number of detected top-5 dimers in shared and species-specific circRNA loci as well as their enrichment after correction for co-occurrence in multiple RVCs (see Materials and methods) are shown. Loci were normalised by the number of detected genes in each category before calculating the enrichment of dimers in shared over species-specific loci. The number of parental genes in both categories is shown below the species name. For mouse, only the top-3 dimers, which are outside the 95% frequency quantile, are shown (see Materials and methods). For rhesus, the analysis could only be done on a subset of genes due to lifting uncertainties between the rheMac2 and the rheMac3 genome (see Materials and methods). [file elife-67991-supp11.docx]

###### **Supplementary File 11: Frequency and enrichment of top-5 dimers in shared and species-specific circRNA loci.**

**Supplementary File 11.** The total number of detected top-5 dimers in shared and species-specific circRNA loci as well as their enrichment after correction for co-occurrence in multiple RVCs (see **Material and Methods**) are shown. Loci were normalized by the number of detected genes in each category before calculating the enrichment of dimers in shared over species-specific loci. The number of parental genes in both categories is shown below the species name. For mouse, only the top-3 dimers, which are outside the 95% frequency quantile, are shown (see **Material and Methods**). For rhesus, the analysis could only be done on a subset of genes due to lifting uncertainties between the rheMac2 and the rheMac3 genome (see **Material and Methods**).

| **Species** | **Dimer** | **Shared loci** | **Species-specific loci** | **Enrichment** |
| --- | --- | --- | --- | --- |
| **opossum**  *n_shared_ = 224*  *n_species-specific_ = 602* | SINE1_Mdo+SINE1_Mdo | 4,634 | 8,155 | 1.53 |
|  | MAR1a_Mdo+MAR1a_Mdo | 535 | 968 | 1.49 |
|  | MAR1a_Mdo+MAR1b_Mdo | 474 | 882 | 1.45 |
|  | SINE1_Mdo+SINE1a_Mdo | 371 | 659 | 1.51 |
|  | MAR1b_Mdo+MAR1b_Mdo | 154 | 276 | 1.50 |
| **mouse**  *n_shared_ = 76*  *n_species-specific_ = 213* | B1_Mus1+B1_Mus2 | 275 | 438 | 1.76 |
|  | B2_Mm2+B2_Mm2 | 268 | 334 | 2.25 |
|  | B1_Mus1+B1_Mus1 | 162 | 274 | 1.66 |
| **rat**  *n_shared_ = 80*  *n_species-specific_ = 260* | ID_Rn1+ID_Rn2 | 184 | 457 | 1.31 |
|  | BC1_Rn+ID_Rn2 | 113 | 248 | 1.49 |
|  | ID_Rn1+ID_Rn1 | 111 | 273 | 1.32 |
|  | BC1_Rn+ID_Rn1 | 108 | 273 | 1.29 |
|  | ID_Rn2+ID_Rn2 | 95 | 224 | 1.38 |
| **rhesus**  *n_shared_ = 38*  *n_species-specific_ = 86* | AluSx+AluSz | 33 | 38 | 1.99 |
|  | AluY+AluYRa1 | 32 | 37 | 1.93 |
|  | AluSx+AluYRa1 | 27 | 21 | 2.86 |
|  | AluSx+AluSx1 | 26 | 35 | 1.68 |
|  | AluSx1+AluSz | 26 | 32 | 1.81 |
| **human**  *n_shared_ = 169*  *n_species-specific_ = 811* | AluSx+AluSx1 | 278 | 980 | 1.36 |
|  | AluSx1+AluY | 274 | 883 | 1.49 |
|  | AluSx+AluY | 269 | 806 | 1.60 |
|  | AluSx1+AluSz | 259 | 958 | 1.30 |
|  | AluSx+AluSz | 257 | 941 | 1.31 |
