# Supplementary material for: Circular RNA repertoires are associated with evolutionarily young transposable elements
Source: eLife. 2021 Sep 20;10:e67991. doi: 10.7554/eLife.67991 (PMC8516420; doi:10.7554/eLife.67991)
Supplement: Transparent reporting form [file elife-67991-transrepform.pdf]

eLife

Westbrook Centre
Milton Road
Cambridge CB4 1YG, UK

P 01223 855340
W elifesciences.org
T @elife

eLife's transparent reporting form

We encourage authors to provide detailed information *within their submission* to facilitate the interpretation and replication of experiments. Authors can upload supporting documentation to indicate the use of appropriate reporting guidelines for health-related research (see EQUATOR Network), life science research (see the BioSharing Information Resource), or the ARRIVE guidelines for reporting work involving animal research. Where applicable, authors should refer to any relevant reporting standards documents in this form.

If you have any questions, please consult our Journal Policies and/or contact us: editorial@elifesciences.org.

Sample-size estimation

- You should state whether an appropriate sample size was computed when the study was being designed
- You should state the statistical method of sample size computation and any required assumptions
- If no explicit power analysis was used, you should describe how you decided what sample (replicate) size (number) to use

Please outline where this information can be found within the submission (e.g., sections or figure legends), or explain why this information doesn't apply to your submission:

Supplementary Table 1 lists the samples and replicates used in the study. Per organ and species, three independent samples (= biological triplicates) were used to generate the datasets (untreated and RNase R treated). An exception had to be made for rhesus macaque cerebellum (two samples) and human liver (one sample) due to scarcity of material in both cases. The use of biological triplicates follows the standard study design in the field of evolutionary transcriptomics. Sample size estimations/power calculations, as for conventional experimental designs, do thus no apply to this kind of study.

- You should report how often each experiment was performed
- You should include a definition of biological versus technical replication
- The data obtained should be provided and sufficient information should be provided to indicate the number of independent biological and/or technical replicates
- If you encountered any outliers, you should describe how these were handled
- Criteria for exclusion/inclusion of data should be clearly stated
- High-throughput sequence data should be uploaded before submission, with a private link for reviewers provided (these are available from both GEO and ArrayExpress)

Please outline where this information can be found within the submission (e.g., sections or figure legends), or explain why this information doesn't apply to your submission:

eLife Sciences Publications, Ltd is a limited liability non-profit non-stock corporation incorporated in the State of Delaware, USA, with company number 5030732, and is registered in the UK with company number FC030576 and branch number BR015634 at the address Westbrook Centre, Milton Road, Cambridge CB4 1YG.

All protocols used to generate the biological replicates of this study are described in the Material & Methods. Biological replicates were defined as the "use of distinct samples from different individuals for each tissue" (e.g. three liver samples, from three different mice). All samples came from male species to reduce sex-specific biases, and individuals within and between species were chosen (whenever possible) to have a similar age to reduce age-specific biases (see Supplementary Table 1 for more details). Each biological replicate was further split into two samples of which one was treated with RNase R. This allowed the direct comparison of circRNA enrichment upon RNase R treatment for each sample.
Moreover, all scripts used to produce the main figures and tables of this publication were deposited in the Git Repository circRNA_paperScripts. This Git repository also holds information on how to run the scripts, and links to the underlying data files for the main figures. The custom pipeline developed for the circRNA identification can be found in the Git Repository ncSplice_circRNAdetection.
All high-throughput sequence data are uploaded to GEO under the GEO accession number GSE162152 (reviewer token: mtgxkuosxhgplcb).

eLife Sciences Publications, Ltd is a limited liability non-profit non-stock corporation incorporated in the State of Delaware, USA, with company number 5030732, and is registered in the UK with company number FC030576 and branch number BR015634 at the address Westbrook Centre, Milton Road, Cambridge CB4 1YG.

Statistical reporting

- Statistical analysis methods should be described and justified
- Raw data should be presented in figures whenever informative to do so (typically when N per group is less than 10)
- For each experiment, you should identify the statistical tests used, exact values of N, definitions of center, methods of multiple test correction, and dispersion and precision measures (e.g., mean, median, SD, SEM, confidence intervals; and, for the major substantive results, a measure of effect size (e.g., Pearson's r, Cohen's d)
- Report exact p-values wherever possible alongside the summary statistics and 95% confidence intervals. These should be reported for all key questions and not only when the p-value is less than 0.05.

Please outline where this information can be found within the submission (e.g., sections or figure legends), or explain why this information doesn't apply to your submission:

All statistical methods and bioinformatics software used in the study are described in the Material & Methods. Sample sizes and statistical test results are mentioned in all figure legends, and if applicable, precise values such as confidence intervals or p-values are summarised in the supplementary tables (e.g. Supplementary Tables 5 – 7).
As stated before, all scripts used to produce the main figures and tables of this publication were deposited in the Git Repository circRNA_paperScripts and provide further information on how certain statistical tests or methods were implemented.

(For large datasets, or papers with a very large number of statistical tests, you may upload a single table file with tests, Ns, etc., with reference to sections in the manuscript.)

Group allocation

- Indicate how samples were allocated into experimental groups (in the case of clinical studies, please specify allocation to treatment method); if randomization was used, please also state if restricted randomization was applied
- Indicate if masking was used during group allocation, data collection and/or data analysis

Please outline where this information can be found within the submission (e.g., sections or figure legends), or explain why this information doesn't apply to your submission:

Not applicable to this experimental design.

Additional data files ("source data")

- We encourage you to upload relevant additional data files, such as numerical data that are represented as a graph in a figure, or as a summary table
- Where provided, these should be in the most useful format, and they can be uploaded as "Source data" files linked to a main figure or table
- Include model definition files including the full list of parameters used
- Include code used for data analysis (e.g., R, MatLab)
- Avoid stating that data files are "available upon request"

Please indicate the figures or tables for which source data files have been provided:

eLife Sciences Publications, Ltd is a limited liability non-profit non-stock corporation incorporated in the State of Delaware, USA, with company number 5030732, and is registered in the UK with company number FC030576 and branch number BR015634 at the address Westbrook Centre, Milton Road, Cambridge CB4 1YG.

All scripts used to produce the main figures and tables of this publication were deposited in the Git Repository circRNA_paperScripts. This Git repository holds information on how to run the scripts, and links to the underlying data files for the main figures as these data files were too large for being uploaded with the submission itself. All scripts (mainly written in R and Ruby) are documented and explain the required data input, how they work, have to be run and the main output files produced.

eLife Sciences Publications, Ltd is a limited liability non-profit non-stock corporation incorporated in the State of Delaware, USA, with company number 5030732, and is registered in the UK with company number FC030576 and branch number BR015634 at the address Westbrook Centre, Milton Road, Cambridge CB4 1YG.
